# Mental health framework: coronavirus pandemic in post-Katrina New Orleans

**DOI:** 10.5249/jivr.vo112i2.1538

**Published:** 2020-07

**Authors:** Denese O. Shervington, Lisa Richardson

**Affiliations:** ^*a*^ Charles Drew R. University of Medicine and Science, Los Angles, California, U.S.A.; ^*b*^ Institute of Women and Ethnic Studies, Los Angles, California, U.S.A.

**Keywords:** Racial minorities, Chronic adversity, Acute shocks, Disaster mental health framework

## Abstract

The United Nations Office of Disaster Risk Reduction defines disaster risk as the “likelihood of loss of life, injury or destruction and damage from a disaster in a given period, and a product of the complex interactions that generate conditions of exposure, vulnerability and hazard”. Racial and ethnic minorities in the United States have been shown to have increased vulnerability and risk to disasters due to links between racism, vulnerability, and economic power, based on disadvantage related to different disaster stages: 1) reduced perception of personal disaster risk; 2) lack of preparedness; 3) reduced access and response to warning systems; 4) increased physical impacts due to substandard housing; 5) likelihood of poorer psychological outcomes; 6) cultural insensitivity on the part of emergency workers; 7) marginalization, lower socio-economic status, and less familiarity with support resources leading to protracted recovery; and 8) diminished standard of living, job loss, and exacerbated poverty during reconstruction and community rebuilding. Moreover, given that psychiatric morbidity is predictable in populations exposed to disasters, mental health and psychosocial support programs should increasingly become a standard part of a humanitarian response. In the crisis and immediate recovery phase of disasters, the focus should be on making survivors feel safe and giving them assistance in decreasing their anxiety by addressing their basic needs and welfare. So, it is critical that governmental institutions, business, and non-profit organizations proactively find mechanisms to work collaboratively and share resources. Special attention and extra resources must be directed towards vulnerable and marginalized populations. In this editorial we share lessons learned from experiencing disproportionate impact of health crisis and advocate for the notion that recovery efforts must address trauma at individual, interpersonal and community levels, and be based in a healing justice framework.

## The Problem

In December 2019, increasing numbers of people in the province of Wuhan, China began seeking medical care for acute respiratory and flu-like symptoms. The novel coronavirus, COVID-19, was found to be the causative agent. Due to the rapid human-to-human spread locally and eventual global spread, the World Health Organization declared COVID-19 a pandemic on March 11, 2020. Since then, many countries have encouraged and/or required varying degrees of public health prevention activities – restricted travel, social distancing, frequent hand washing, coughing into elbows, not touching the face, and staying at home if feeling sick.

New Orleans’ annual Mardi Gras celebration culminated in the last two weeks of February 2020, with over 1.4 million visitors in the city. The first case of COVID-19 was reported on March 9, and the first death was reported on March 14. On Friday, March 20, after at least 14 people died due to COVID-related illnesses, the Mayor issued a stay-at-home order asking residents to avoid large crowds and to go outside only for essential services. According to the Louisiana Department of Health Office of Public Health, as of March 31, 5237 cases had been reported in Louisiana, with 239 deaths. Eighteen hundred and thirty-four cases were reported in Orleans Parish, of which 101 people have died. 

The city of New Orleans has become one of the national epicenters of the COVID-19 pandemic. An article appearing on March 27 in The Atlantic entitled, “Watch New Orleans: With the country’s attention turned north, the coronavirus pandemic is exploding in Louisiana,” journalist Van Newkirk paints an alarming picture of the public health crisis. Approximately 1% of the U.S. population lives in Louisiana, but the state reports 7% of all COVID-19 deaths, 7% of all hospitalizations, and 3% of all positive tests nationwide. The Louisiana Department of Health and Hospitals statistics indicate that Orleans and Jefferson parishes have the highest coronavirus death rates in the country. In Orleans Parish, there are 14.6 COVID-19 deaths for every 100,000 residents, and 5.5 for Jefferson Parish. By comparison, both parishes have significantly higher death rates than King County, in Seattle, which reports 4.8 deaths for every 100,000 residents and New York City that reports 4.4 deaths for every 100,000 residents.

**The Historical and Disproportionate Impacts of Disasters**

The United Nations Office of Disaster Risk Reduction defines disaster risk as the “likelihood of loss of life, injury or destruction and damage from a disaster in a given period, and a product of the complex interactions that generate conditions of exposure, vulnerability and hazard”.^1^ Racial and ethnic minorities in the United States have been shown to have increased vulnerability and risk to disasters due to links between racism, vulnerability, and economic power, based on disadvantage in each of the eight disaster stages: 1) reduced perception of personal disaster risk; 2) lack of preparedness; 3) reduced access and response to warning systems; 4) increased physical impacts due to substandard housing; 5) likelihood of poorer psychological outcomes; 6) cultural insensitivity on the part of emergency workers; 7) marginalization, lower socio-economic status, and less familiarity with support resources leading to protracted recovery; and 8) diminished standard of living, job loss, and exacerbated poverty during reconstruction and community rebuilding.^2^

The New Orleans Data Center has highlighted that the disparate severity of COVID-19 in Orleans Parish can be attributed to a long history of racial inequities and socioeconomic disadvantages. The report went on to highlight various inequities, inequalities, and disparities, due largely to race and socio-economic status: 1) compared to other hot-spots, New Orleans has higher poverty rates and lower average incomes; 2) nearly 1 in 5 New Orleans households do not have access to a vehicle, making drive-up testing close to impossible; 3) twenty two percent of New Orleanians have no access to internet, including smart phones; 4) New Orleans adults suffer from high blood pressure, diabetes, chronic kidney disease, and other preexisting conditions at rates higher than in Seattle, New Rochelle, or New York City.^3^ The impact of COVID-19 is further amplified by the well-documented legacy of institutional racism and stark economic and health inequities, including limited access to quality healthcare. The high prevalence of the pre-existing conditions that put people at risk for serious complications from the coronavirus reflect the outcomes associated with negative social determinants of health.

**Disaster Mental Health**

Disasters reflect an encounter between a hazardous force (in this case the coronavirus) and a human population in harm’s way which, within the ecological context, can create demands that exceed the coping capacity of the affected community.^4^ A disaster’s forces of harm (loss and change) are a complex interplay of the interrelationship and interdependence of social and ecological factors–the individual/family context, the community context, and the societal/structural context. New Orleans, nearly 15 years ago, experienced a major disaster.

On August 29, 2005, Hurricane Katrina struck America’s Gulf Coast. Katrina was the deadliest hurricane in seven decades to hit the United States, bringing severe winds and record rainfall into New Orleans for a 24-hour period. Two days of intense storm surge damaged the city’s pumping system, rendering it incapable of draining the rising water as major floodwalls failed along multiple city waterways. As a result, 80% of the city flooded, and homes, communities, and the urban infrastructure were destroyed. For weeks, the city was submerged in floodwaters as high as five meters, which resulted in extensive structural damage. Service delivery was brought to a standstill, and emergency and rescue efforts were severely hampered. More than 500,000 people were evacuated, and a minimum of 1800 people died from storm-related causes.^5,6^ In 2012, the National Weather Service estimated that there was at least $108 billion dollars in property damage from Hurricane Katrina, making it the costliest natural disaster in U.S. history at that time.

A 2005 survey conducted two months post-Katrina by the Centers for Disease Control and Prevention and the Louisiana Office of Mental Health found that 45% of those sampled were suffering from PTSD.^7^ A longitudinal survey of adult Gulf Coast residents (n=1043) who were directly affected by Hurricane Katrina found the prevalence of PTSD six months post-Katrina was twice as high as the prevalence estimates for the population in the years prior to the hurricane.^8^ In this study, poor, racial and ethnic minorities and those with fewer years of formal education more commonly reported stressful experiences post-evacuation.

Notably, PTSD symptomatology was most common among those who lived in New Orleans–49.6% of these respondents reported nightmares, 52.8% reported being jumpier or startled more easily, and 79.4% reported being more irritable or angry than usual. The follow-up survey conducted a year later found that anxiety or mood disorders persisted and increased slightly from 30.7% to 33.9%, and the prevalence of PTSD had almost doubled among those residents who remained displaced. Similarly, suicidality was significantly higher with regard to suicidal ideation and suicide plans.

A longitudinal study of low-income African American mothers pre- and post-Katrina found that even though symptoms of Post-Traumatic Stress Disorder (PTSD) declined over time after the hurricane, they remained high–33% of the sample showed scores suggesting continued symptoms of PTSD 43-54 months after the hurricane. The study also found that there was in increase in psychological distress from 26% pre-Katrina to 30% 43-54 months after the hurricane. Home damage was an important predictor of chronic symptoms of PTSD.^9^ These data differ from the typical post-disaster circumstances where mental disorders significantly decrease with time and up to 50% typically resolve within a year.^10^ For example, the doubling of PTSD levels noted two months after the September 11 attacks returned to baseline 4-6 months later.^11^ These data illustrate the more severe adverse psychological effects of Hurricane Katrina compared to other disasters and emphasize the disproportionate mental health impacts of disasters on socio-economically disadvantaged racial and ethnic minority groups.

A survey conducted by the Institute of Women and Ethnic Studies (IWES) in 2007 with 80 adult African American Katrina evacuees who had returned to New Orleans within two years of the hurricane revealed that 72% reported irritability and depression, 60% reported appetite changes, 54% reported sleep disturbances, and 50% reported difficulty concentrating. Regarding symptoms of PTSD, 20% reported flashbacks, 33% reported avoidance of stimuli, and 27% reported startling more easily.^12^ Since 2012, IWES has assessed youth for symptoms of PTSD and depression based on criteria from the American Psychiatric Association’s Diagnostic and Statistical Manual Fifth Edition^13^ and screened youth for exposure to violence and worries about their basic needs. Close to 15 years post-Katrina, psychosocial screenings show an extremely high prevalence of traumatic stress and mental health disorder symptoms among youth aged 11-19 in New Orleans. Of the 5827 youth surveyed thus far, 18% endorse symptoms of depression; 35% endorse symptoms of lifetime PTSD with 21% endorsing symptoms of current PTSD; 34% report exposure to domestic violence; 51% report having lost a family member or someone close to murder; and 33% worry about not being loved. Of note, the national prevalence rate for adolescent PTSD is 4%^14^ while the rate for depression is 13%.^15^

Unlike disasters that are land-based, visually apparent, and time-bound–hurricanes, fires, earthquakes, tornadoes, flooding, and wind/sand storms—this disaster is silent, unseen, and highly unpredictable, and at this point there is no way of determining when the pandemic will end. This uncertainty and unpredictability about the spread of COVID-19 and its impact has created significant emotional distress. A recent poll by the American Psychiatric Association conducted March 18-19, 2020 found that a significant number of Americans (62%) are anxious about the possibility of family and loved ones contracting COVID-19. The survey also found that 36% of Americans report that the coronavirus is having a serious impact on their mental health, with 59% reporting that the virus is having a significant impact on their day-to-day lives. Over 50% are worried about running out of food, medicine and/or supplies, and 57% worry that the virus will have a significant impact on their finances. It should be anticipated that mental health needs in New Orleans during and after the coronavirus crisis will be significant and may be greater than in other US cities, given higher baseline (Katrina-related) levels of trauma-based conditions. Additionally, as has occurred globally, it is expected that frontline healthcare providers, at greatest risk for contagion from the virus,^16^ will be at great risk of developing unfavorable mental health outcomes.

Those who experience catastrophic events show a wide range of reactions: some suffer only worries and bad memories that fade with emotional support and the passage of time; others are more deeply affected and experience long-term problems—PTSD, depression, generalized anxiety disorders, and substance use disorders are the most common post trauma psychiatric sequelae.^17^ A systematic review of post- traumatic stress disorders following disasters in the past three decades concluded that the burden of PTSD among people exposed to disasters is substantial and is correlated with factors such as socio-demographic and background factors, event exposure, social support, and personality traits.^10^ In a February 2020 blog, the American Psychiatric Association Committee on Psychiatric Dimensions noted that adverse psychological and behavioral responses to infectious disease outbreaks are common and include insomnia, reduced feelings of safety, scapegoating, increased use of alcohol and tobacco, somatic symptoms (physical symptoms) such as lack of energy and general aches and pains), and increased use of medical resources.^18^

**Evidence-based Global and National Mental Health**

Given that psychiatric morbidity is predictable in populations exposed to disasters, mental health and psychosocial support programs should increasingly become a standard part of a humanitarian response.^19^ A global panel of experts on disaster and mass violence identified five key intervention principles that should be used to guide and inform intervention and prevention efforts at the early to midterm stages of the event. These principles are: 1) promote a sense of safety; 2) promote calm; 3) promote a sense of self- and collective efficacy; 4) promote connectedness; and 5) promote hope.^20^ These principles were empirically found to restore social and behavioral functioning after disasters.

In the crisis and immediate recovery phase of disasters, the focus should be on making survivors feel safe and giving them assistance in decreasing their anxiety by addressing their basic needs and welfare.^21^ In the lessons learned after the fireworks disaster in Enschede, Netherlands, soon after a disaster, survivors must be helped to regain their autonomy, reserving clinical psychiatric help for those exhibiting dissociative symptoms or those with prolonged mental health symptoms that showed no improvement after two months.^22^

Similarly, the World Health Organization’s guidance (June 2019) regarding mental health in emergencies advises.^23^

• Strengthen community self-help and social support

• Offer psychological first aid–first-line emotional and practical support

• Assure basic mental health care for priority conditions (e.g. depression, psychotic disorders, epilepsy, substance use disorder, etc.) is provided at every health-care facility by trained and supervised general health staff)

• Provide psychological interventions offered by specialists, trained in trauma-focused approaches, for people impaired by prolonged distress

• Protect and promote the rights of people with severe mental health conditions and psychosocial disabilities

• Create links and referral mechanisms between mental health specialists, general health-care providers, community-based support, and other services (e.g. schools, social services, and emergency relief services such as those providing food, water, and housing/shelter)

The National Child Traumatic Stress Network and the National Center recommend Psychological First Aid (PFA)^24^ for PTSD when providing early assistance within days or weeks following an event. PFA is an evidence-informed modular approach to help children, adolescents, adults, and families in the immediate aftermath of disaster and terrorism. PFA is designed to reduce the initial distress caused by traumatic events and to foster short-and long-term adaptive functioning and coping. The core objectives are listed in Table 1.

**Table 1:** Psychological First Aid Core Objectives.

Action	Goal
1. Contact and Engagement	To respond to contacts initiated by survivors or to initiate contacts in a non-intrusive, compassionate, and helpful manner.
2. Safety and Comfort	To enhance immediate and ongoing safety and provide physical and emotional comfort.
3. Stabilization (if needed)	To calm and orient emotionally overwhelmed or disoriented survivors.
4. Information Gathering: Current Needs and Concerns	To identify immediate needs and concerns, gather additional information, and tailor Psychological First Aid interventions.
5. Practical Assistance	To offer practical help to survivors in addressing immediate needs and concerns.
6. Practical Assistance	To offer practical help to survivors in addressing immediate needs and concerns.
7. Information on Coping	To provide information about stress reactions and coping to reduce distress and promote adaptive functioning
8. Linkage with Collaborative Services	To link survivors with available services needed at the time or in the future.

**Recommendations for New Orleans Population-level Mental Health**

In response to the current viral pandemic, the following strategic activities are being recommended based on local adaption to the aforementioned global and national frameworks, as well as lessons learned from Hurricane Katrina:

**Immediate Crisis**

1. Support organizations/institutions addressing basic needs (food, housing, finances) and access to healthcare

2. Promote physical safety in the population at large

a. Provide education regarding coronavirus transmission and steps to prevent transmission and dispel myths

b. Provide education regarding when to seek testing and/or hospital services

3. Promote psychological safety through virtual/digital and social media; traditional media–print, radio, TV, and billboards:

a. Provide normalizing psycho-education regarding fear, anxiety, and mood disturbances (normal response to the threat of harm from virus)

b. Create virtual connection and community to enhance individual and collective efficacy, interpersonal learning, and hopefulness/optimism

c. Teach calming and coping mechanisms for the general population and targeted populations (medical providers, teachers, new parents, CBOs)

i. Deep breathing

ii. Mindfulness meditation

iii. Overall wellness and self-care affirmations

4. Assure access to tele-health resources and medication for residents with existing or newly acquired serious mental health disorders

5. Conduct a rapid assessment of community knowledge, attitudes, beliefs, and actions regarding coronavirus.

**Recovery Phase**

1. Prepare mental health systems (public and private) to provide culturally-proficient trauma-based services for children, adolescents, and adults

2. Conduct research to assess level of trauma-based disorders

3. Conduct trainings at multiple levels in educational system to assist schools in being able to realize, recognize, and respond to increased levels of trauma conditions in students – i.e. assist schools in adopting trauma-informed and restorative practices

4. Increase access to mental health services–community and school-based

## Conclusion

With Hurricane Katrina, widespread assessment of disaster response and recovery efforts emphasized the lack of effective leadership within the Federal Emergency Management Agency which, under the authorization of the U.S. Department of Homeland Security, coordinates communications across federal agencies in response to disasters. The inadequate rescue and failed governmental response to the disaster was decried by human rights experts as “shocking, a gross violation of human rights”.^25^ A select bipartisan committee of the U.S. House of Representatives investigating the hurricane cited failures at all levels of government. The report, “A Failure of Initiative,” noted that medical care and evacuations suffered from a lack of advance preparations, inadequate communications, and inadequate coordination, and that the failure of complete evacuations led to preventable deaths, great suffering, and further delays in relief.^26^

The impact that the virus will have on the city will inevitably conjure memories of Katrina for many New Orleanians. COVID-19 will affect every aspect of life. Its predicted force is akin to a tsunami: a devastating eruption generating a series of progressive waves that sweep across the land in ever-widening circles. So as to never repeat the failures of Hurricane Katrina, it is critical that governmental institutions, business, and non-profit organizations proactively find mechanisms to work collaboratively and share resources. Special attention and extra resources must be directed towards vulnerable and marginalized populations. For example, children that were born into and lived through the aftermath of Katrina continue to show emotional distress nearly 15 years after the disaster. The trauma caused by this public health crisis will be carried and embodied for the longest time by the youth that are living through it.

Meaningful action must be taken immediately to mitigate the possible devastating impact of this virus on the youth of New Orleans. Experts are calling for wide-ranging federal action including direct payments to families during this crisis, a national moratorium on rent and eviction, additional support for the homeless, and emergency resources for children in foster care. Vann Newkirk’s March 24, 2020 article in The Atlantic, “The Kids Aren’t All Right”, focuses on the trauma and long-term economic impact that the crisis will have on children.^27^ Newkirk interviewed Bruce Lesley, the president of First Focus on Children, who is advocating for an expansion of the Supplemental Nutrition Assistance Program (SNAP), and Alice Fothergill, who co-authored “Children of Katrina” with Lori Peek. Fothergill spent seven years studying the effects of Katrina on young people and found that existing social disadvantages, in this case poverty and race, fueled an uneven recovery among kids based on their socio-economic circumstances. She noted, “Disasters last a really long time in the lives of children. People are talking about vulnerability, but they are not talking about children at all.”

As Dr. Shervington has advised on billboards throughout the city of New Orleans in the years post-Katrina as youth violence began to spike, ‘Untreated trauma is the underbelly of violence’. The COVID-19 pandemic now grips New Orleans in another disaster. We have learned that response and recovery efforts must address trauma at individual, interpersonal and community levels, and be based in a healing justice framework, as outlined in IWES’ 2019 publication, Healing is the Revolution.^28^

## References

[1] United Nations International Strategy for Disaster Reduction. What is Disaster Risk Reduction? http://www.drdm.gov.sc/wp-content/uploads/2017/05/UNISDR-terminology-2009-eng.pdf, accessed 24 March 2020.

[2] Fothergill A, Maestas EG, Darlington JD (1999). Race, ethnicity and disasters in the United States: A review of the literature. Disasters.

[3] New Orleans Data Center. Monitoring the COVID-19 Pandemic in New Orleans and Louisiana, https://www.datacenterresearch.org/covid-19-data-and-information/covid-19-data/, accessed 24 March 2020.

[4] Ursano RJ, Fullerton CS, Weisaeth L, Raphael B. Textbook of disaster psychiatry. Cambridge University Press; 2007.

[5] Zimmerman K. Hurricane Katrina: Facts, Damage and Aftermath. Live Science, https://www.livescience.com/22522-hurricane-katrina-facts.html. Published 2015, accessed 25 March 2020.

[6] Graduate School of Oceanography. Hurricanes: Science and Society. http://www.hurricanescience.org/history/studies/katrinacase/impacts/, accessed 25 March 2020.

[7] Control CfD (2006). Prevention. Surveillance for illness and injury after Hurricane Katrina--three counties, Mississippi, September 5-October 11, 2005. MMWR: Morbidity and Mortality Weekly Report.

[8] Brewin C, Galea, S, Jones R, Kessler R, King L, Lurie N, et al. Overview of Baseline Survey Results: Hurricane Katrina Community Advisory Group. http://www.npr.org/docu-ments/2006/aug/katrina_mental_health.pdf, accessed 26 March 2020.

[9] Paxson C, Fussell E, Rhodes J, Waters M (2012). Five years later: Recovery from post traumatic stress and psychological distress among low-income mothers affected by Hurricane Katrina. Soc Sci Med.

[10] Neria Y, Nandi A, Galea S (2008). Post-traumatic stress disorder following disasters: a systematic review. Psychol Med.

[11] Galea S, Boscarino JA, Resnick HS, Vlahov D. Mental health in New York City after the September 11 terrorist attacks: results from two population surveys. In: Manderscheid, Henderson M, eds. Mental Health, United States 2001. Washington DC: Government Printing Office; 2002.

[12] Shervington D (2007). No One is Coming to Save Us: Coping with the Stressful Aftermath of Katrina. Journal of the National Medical Association.

[13] American Psychiatric Association. Diagnostic and Statistical Manual of Mental Disorders. Fifth Edition ed: Arlington, VA; 2013.

[14] Kessler RC, Avenevoli S, Costello EJ, Green JG, Gruber MJ, Georgiades K (2012). Prevalence, persistence, and sociodemographic correlates of DSM-IV disorders in the National Comorbidity Survey Replication Adolescent Supplement. Arch Gen Psychiatry.

[15] National Institute of Mental Health. Depression. https://www.nimh.nih.gov/health/index.shtml, accessed 24 March 2020.

[16] Lai J, Ma S, Wang Y, Cai Z, Hu J, Wei N (2020). Factors Associated with Mental Health Outcomes Among Health Care Workers Exposed to Coronavirus Disease. JAMA Netw Open.

[17] Galea S, Nandi A, Vlahov D (2005). The epidemiology of post-traumatic stress disorder after disasters. Epidemiol Rev.

[18] American Psychiatric Association. Committee on Psychiatric Dimensions of Disasters. psychiatry.org/news-room/apa-blogs/apa-blog2020, accessed 24 February 2020.

[19] Tol WA, Ommeren M (2012). van. Evidence-based mental health and psychosocial support in humanitarian settings: Gaps and opportunities. EvidenceBased Mental Health.

[20] Hobfoll SE, Watson P, Bell CC, Bryant RA, Brymer MJ, Friedman MJ, et al. Five Essential Elements of Immediate and Mid-Term Mass Trauma Intervention: Empirical Evidence. Psychiatry. 2007 Winter;70(4):283-315; discussion 316-69. 10.1521/psyc.2007.70.4.283 18181708

[21] McFarlane AC, Williams R (2012). Mental Health Services Required after Disasters: Leaning from the lasting Effects of Disasters. Depress Res Treat.

[22] Bosman F, Bakker H, Fullilove MT (2013). Mental Health Center in Post-Disaster Recovery: Ten-Year Retrospective of Mediant's Work in Enschede, Netherlands. International Journal of Mental Health.

[23] World Health Organization. https://www.who.int/news-room/fact-sheets/detail/mental-health-in-emergencies, accessed 24 March 2020.

[24] Brymer M, Jacobs A, Layne C, Pynoos R, Ruzek J, Steinberg A, et al. (National Child Traumatic Stress Network and National Center for PTSD), Psychological First Aid: Field Operations Guide, 2nd Edition, 2006.

[25] Warren C. Poverty Expert finds New Orleans ‘shocking’. In. Advocate, October 29. Baton Rouge 2005.

[26] Select Bipartisan Committee to Investigate the Preparation for and Response to Hurricane Katrina. A failure of initiative. U.S. Government Printing Office (Published 2006), https://www.govinfo.gov/content/pkg/CRPT-109hrpt377/pdf/CRPT-109hrpt377.pdf, accessed 24 March 2020.

[27] Newkirk V. The Kids Aren’t All Right. The Atlantic. https://www.theatlantic.com/magazine/archive/2010/07/the-kids-arent-all-right/308162/, accessed March 2020.

[28] Shervington D. Healing Is the Revolution. Institute of Women and Ethnic Studies, New Orlean, Louisiana; 2018.

